# Poly[(μ_3_-4-carb­oxy­pyridine-3-carboxyl­ato-κ^3^ *N*:*O* ^3^:*O* ^4^)(triphenyl­phosphine-κ*P*)silver(I)]

**DOI:** 10.1107/S1600536810033350

**Published:** 2010-08-25

**Authors:** Omid Sadeghi, Mostafa M. Amini, Seik Weng Ng

**Affiliations:** a Department of Chemistry, General Campus, Shahid Beheshti University, Tehran 1983963113, Iran; b Department of Chemistry, University of Malaya, 50603 Kuala Lumpur, Malaysia

## Abstract

In the title 1:1 silver(I) 4-carb­oxy­pyridine-3-carboxyl­ate adduct with triphenyl­phosphine, [Ag(C_7_H_4_NO_4_)(C_18_H_15_P)]_*n*_, the carboxyl­ate anion bridges the phosphine-coordinated Ag atoms through its N and O atoms, generating a coordination polymer forming layers in the *bc* plane. The Ag atom exists in a distorted tetra­hedral geometry. The H atom of the carboxyl­ate is midway between two O atoms of the two carboxyl groups, thus forming a strong intra­molecular hydrogen bond.

## Related literature

For the synthesis of the silver reactant used in the synthesis, see: Hanna & Ng (1999 ▶); Ng & Othman (1997 ▶). For a related structure, see: Drew *et al.* (1971 ▶).
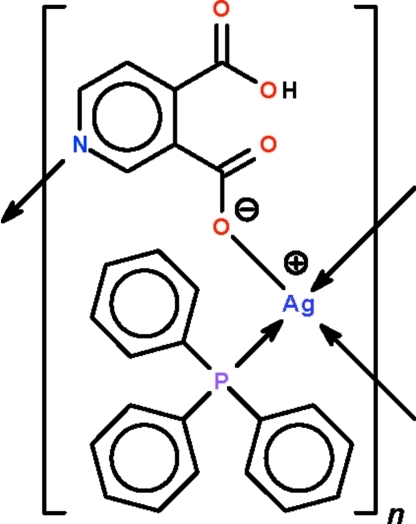


## Experimental

### 

#### Crystal data

[Ag(C_7_H_4_NO_4_)(C_18_H_15_P)]
*M* *_r_* = 536.25
Monoclinic, 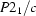

*a* = 14.2472 (7) Å
*b* = 10.2431 (5) Å
*c* = 16.3146 (8) Å
β = 115.206 (1)°
*V* = 2154.18 (18) Å^3^
*Z* = 4
Mo *K*α radiation
μ = 1.04 mm^−1^
*T* = 100 K
0.35 × 0.30 × 0.15 mm

#### Data collection

Bruker SMART APEX diffractometer
Absorption correction: multi-scan (*SADABS*; Sheldrick, 1996 ▶) *T*_min_ = 0.712, *T*_max_ = 0.859
13432 measured reflections
4942 independent reflections
4587 reflections with *I* > 2σ(*I*)
*R*_int_ = 0.019

#### Refinement

*R*[*F* ^2^ > 2σ(*F* ^2^)] = 0.020
*wR*(*F* ^2^) = 0.055
*S* = 1.05
4942 reflections
293 parameters
H atoms treated by a mixture of independent and constrained refinement
Δρ_max_ = 0.43 e Å^−3^
Δρ_min_ = −0.48 e Å^−3^

### 

Data collection: *APEX2* (Bruker, 2009 ▶); cell refinement: *SAINT* (Bruker, 2009 ▶); data reduction: *SAINT*; program(s) used to solve structure: *SHELXS97* (Sheldrick, 2008 ▶); program(s) used to refine structure: *SHELXL97* (Sheldrick, 2008 ▶); molecular graphics: *X-SEED* (Barbour, 2001 ▶); software used to prepare material for publication: *publCIF* (Westrip, 2010 ▶).

## Supplementary Material

Crystal structure: contains datablocks global, I. DOI: 10.1107/S1600536810033350/bt5322sup1.cif

Structure factors: contains datablocks I. DOI: 10.1107/S1600536810033350/bt5322Isup2.hkl

Additional supplementary materials:  crystallographic information; 3D view; checkCIF report

## Figures and Tables

**Fig. 1.**
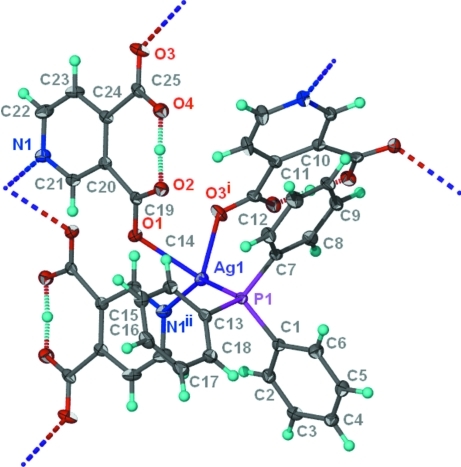
Anisotropic displacement ellipsoid plot (Barbour, 2001) of a portion of the title polymer; ellipsoids are drawn at the 70% probability level and H atoms are of arbitrary radius. Symmetry transformations are given in Table 1.: i = 1 - x, 1 - y, 1 - z; ii = 3/2 - x, y - 1/2, 3/2 - z.

## supplementary crystallographic information

### Comment

We have used bis(silver acetate^.^2triphenylphosphine) monohydrate sesquiethanol (Hanna & Ng, 1999; Ng & Othman, 1997) as a template in the synthesis of triphenylphosphine adducts of other silver carboxylates; the silver carboxylates themselves cannot be synthesized directly by the reaction of a silver salt with the carboxylate anion as the reaction invariably leads to the formation of some insoluble gray material.

The crystal structure of the silver(II) derivative of the monobasic 3-carboxypyridyl-4-carboxylate anion was reported a long time ago (Drew *et al.*, 1971); the silver atom is *N*,*O*-chelated by two anions in an approximate square-planar enviroment.

The silver(I) 3-carboxypyridyl-4-carboxylate–triphenylphosphine adduct (Scheme I) exists as a polymeric compound (Fig. 1) in which the anion bridges adjacent silver atoms through one carboxyl group and the pyridyl N atom (Fig. 2). The diffraction measurements are of a sufficiently high quality for the acid H atom to be refined; the refinement places this atom mid-way between the O atoms of the two carboxyl O atoms, at a distance of 1.20 (4) Å. The O···H···O interaction is an intramolecular hydrogen bond. The pyridyl ring and the carboxyl –CO_2_ unit that is engaged in Ag coordination enclose a dihedral angle of  14.3 (2) ° whereas the free carboxyl group encloses a dihedral angle of 15.2 (3) ° with the pyridyl ring; such minimal twist probably locks the acid H atom in its place.

### Experimental

Silver acetate (1 mmol, 0.17 g) and triphenylphosphine (2 mmol, 0.53 g) were heated in ethanol (50 ml) until the reactants dissolved completely. Gray insoluble material was removed by filtration and the solvent removed to yield bis(silver acetate^.^2triphenylphosphine) monohydrate sesquiethanol (Hanna & Ng, 1999; Ng & Othman, 1997).

The adduct (0.5 mmol, 0.69 g) and 3,4-pyridinedicarboxylic acid (1 mmol, 0.17 g) were placed in a convection tube; the tube was filled with a 1:1 methanol/ethanol mixture and kept at 333 K. Colorless crystals were collected after 3 days (m.p. > 550 K).

### Refinement

Hydrogen atoms bonded to C were placed in calculated positions (C–H 0.95 Å) and were included in the refinement in the riding model approximation, with *U*(H) set to 1.2*U*_eq_(C). The carboxylic H-atom  was freely refined.

### Crystal data

**Table:**

[Ag(C_7_H_4_NO_4_)(C_18_H_15_P)]	*F*(000) = 1080
*M**_r_* = 536.25	*D*_x_ = 1.653 Mg m^−^^3^
Monoclinic, *P*2_1_/*c*	Mo *K*α radiation, λ = 0.71073 Å
Hall symbol: -P 2ybc	Cell parameters from 9293 reflections
*a* = 14.2472 (7) Å	θ = 2.4–28.3°
*b* = 10.2431 (5) Å	µ = 1.04 mm^−^^1^
*c* = 16.3146 (8) Å	*T* = 100 K
β = 115.206 (1)°	Block, colorless
*V* = 2154.18 (18) Å^3^	0.35 × 0.30 × 0.15 mm
*Z* = 4	

### Data collection

**Table:**

Bruker SMART APEX diffractometer	4942 independent reflections
Radiation source: fine-focus sealed tube	4587 reflections with *I* > 2σ(*I*)
graphite	*R*_int_ = 0.019
ω scans	θ_max_ = 27.5°, θ_min_ = 1.6°
Absorption correction: multi-scan (*SADABS*; Sheldrick, 1996)	*h* = −18→18
*T*_min_ = 0.712, *T*_max_ = 0.859	*k* = −10→13
13432 measured reflections	*l* = −21→20

### Refinement

**Table:**

Refinement on *F*^2^	Primary atom site location: structure-invariant direct methods
Least-squares matrix: full	Secondary atom site location: difference Fourier map
*R*[*F*^2^ > 2σ(*F*^2^)] = 0.020	Hydrogen site location: inferred from neighbouring sites
*wR*(*F*^2^) = 0.055	H atoms treated by a mixture of independent and constrained refinement
*S* = 1.05	*w* = 1/[σ^2^(*F*_o_^2^) + (0.0292*P*)^2^ + 1.0816*P*] where *P* = (*F*_o_^2^ + 2*F*_c_^2^)/3
4942 reflections	(Δ/σ)_max_ = 0.001
293 parameters	Δρ_max_ = 0.43 e Å^−^^3^
0 restraints	Δρ_min_ = −0.48 e Å^−^^3^

### Fractional atomic coordinates and isotropic or equivalent isotropic displacement parameters (Å^2^)

**Table:**

	*x*	*y*	*z*	*U*_iso_*/*U*_eq_	
Ag1	0.618798 (9)	0.207715 (12)	0.474714 (8)	0.01345 (5)	
P1	0.76750 (3)	0.10818 (4)	0.47440 (3)	0.01115 (8)	
O1	0.62846 (9)	0.44669 (12)	0.47885 (7)	0.0165 (2)	
O2	0.69543 (9)	0.48204 (12)	0.38079 (8)	0.0166 (2)	
O3	0.53857 (9)	0.75596 (13)	0.16600 (8)	0.0193 (2)	
O4	0.66107 (9)	0.61847 (12)	0.25149 (8)	0.0178 (2)	
H4	0.679 (3)	0.554 (4)	0.318 (2)	0.095 (12)*	
N1	0.46880 (10)	0.77905 (13)	0.43864 (10)	0.0148 (3)	
C1	0.78294 (12)	−0.06194 (15)	0.50936 (10)	0.0127 (3)	
C2	0.73968 (12)	−0.10399 (16)	0.56721 (11)	0.0146 (3)	
H2	0.6994	−0.0453	0.5842	0.018*	
C3	0.75513 (13)	−0.23085 (17)	0.60006 (11)	0.0160 (3)	
H3	0.7260	−0.2587	0.6398	0.019*	
C4	0.81319 (13)	−0.31702 (16)	0.57480 (11)	0.0158 (3)	
H4A	0.8246	−0.4036	0.5979	0.019*	
C5	0.85461 (14)	−0.27720 (17)	0.51589 (12)	0.0175 (3)	
H5	0.8930	−0.3372	0.4977	0.021*	
C6	0.84020 (13)	−0.14963 (17)	0.48331 (11)	0.0166 (3)	
H6	0.8693	−0.1223	0.4434	0.020*	
C7	0.77577 (12)	0.10772 (15)	0.36598 (10)	0.0122 (3)	
C8	0.69787 (12)	0.04132 (16)	0.29445 (11)	0.0159 (3)	
H8	0.6462	−0.0053	0.3047	0.019*	
C9	0.69546 (13)	0.04295 (17)	0.20850 (11)	0.0176 (3)	
H9	0.6430	−0.0036	0.1604	0.021*	
C10	0.76977 (13)	0.11260 (17)	0.19307 (11)	0.0189 (3)	
H10	0.7670	0.1157	0.1339	0.023*	
C11	0.84788 (14)	0.17757 (18)	0.26345 (12)	0.0211 (4)	
H11	0.8992	0.2241	0.2527	0.025*	
C12	0.85148 (13)	0.17507 (17)	0.35027 (11)	0.0173 (3)	
H12	0.9055	0.2193	0.3986	0.021*	
C13	0.88689 (12)	0.18607 (16)	0.55374 (11)	0.0132 (3)	
C14	0.89652 (13)	0.32173 (17)	0.54761 (11)	0.0155 (3)	
H14	0.8419	0.3704	0.5029	0.019*	
C15	0.98636 (13)	0.38491 (17)	0.60728 (11)	0.0185 (3)	
H15	0.9934	0.4765	0.6024	0.022*	
C16	1.06569 (13)	0.31447 (18)	0.67385 (12)	0.0191 (3)	
H16	1.1267	0.3578	0.7145	0.023*	
C17	1.05556 (13)	0.18084 (18)	0.68081 (11)	0.0186 (3)	
H17	1.1096	0.1328	0.7266	0.022*	
C18	0.96635 (12)	0.11650 (17)	0.62095 (11)	0.0158 (3)	
H18	0.9598	0.0249	0.6261	0.019*	
C19	0.63603 (11)	0.51340 (15)	0.41887 (10)	0.0127 (3)	
C20	0.57237 (11)	0.63798 (15)	0.39057 (10)	0.0119 (3)	
C21	0.52976 (12)	0.67522 (16)	0.44994 (11)	0.0130 (3)	
H21	0.5453	0.6228	0.5022	0.016*	
C22	0.44636 (13)	0.85140 (18)	0.36360 (12)	0.0191 (3)	
H22	0.4028	0.9256	0.3536	0.023*	
C23	0.48410 (13)	0.82197 (17)	0.30104 (12)	0.0177 (3)	
H23	0.4657	0.8755	0.2489	0.021*	
C24	0.54897 (12)	0.71481 (15)	0.31276 (11)	0.0124 (3)	
C25	0.58450 (12)	0.69438 (16)	0.23706 (11)	0.0140 (3)	

### Atomic displacement parameters (Å^2^)

**Table:**

	*U*^11^	*U*^22^	*U*^33^	*U*^12^	*U*^13^	*U*^23^
Ag1	0.01300 (7)	0.01372 (7)	0.01545 (7)	0.00322 (4)	0.00782 (5)	0.00133 (4)
P1	0.01137 (17)	0.01234 (19)	0.01010 (18)	0.00181 (14)	0.00492 (15)	0.00062 (14)
O1	0.0218 (6)	0.0127 (6)	0.0151 (6)	0.0016 (4)	0.0081 (5)	0.0017 (4)
O2	0.0175 (5)	0.0186 (6)	0.0147 (5)	0.0052 (5)	0.0077 (5)	0.0007 (5)
O3	0.0168 (6)	0.0290 (7)	0.0106 (5)	−0.0030 (5)	0.0043 (5)	0.0032 (5)
O4	0.0212 (6)	0.0192 (6)	0.0168 (6)	0.0025 (5)	0.0118 (5)	0.0012 (5)
N1	0.0136 (6)	0.0163 (7)	0.0156 (7)	0.0025 (5)	0.0074 (5)	0.0031 (5)
C1	0.0123 (7)	0.0135 (7)	0.0110 (7)	0.0007 (6)	0.0037 (6)	0.0004 (6)
C2	0.0143 (7)	0.0170 (8)	0.0134 (7)	0.0018 (6)	0.0067 (6)	−0.0013 (6)
C3	0.0154 (7)	0.0197 (8)	0.0124 (7)	−0.0016 (6)	0.0056 (6)	0.0011 (6)
C4	0.0160 (7)	0.0136 (8)	0.0139 (7)	−0.0004 (6)	0.0026 (6)	0.0007 (6)
C5	0.0184 (8)	0.0162 (8)	0.0188 (8)	0.0035 (6)	0.0088 (7)	−0.0010 (6)
C6	0.0182 (8)	0.0166 (8)	0.0175 (8)	0.0024 (6)	0.0101 (7)	0.0019 (6)
C7	0.0133 (7)	0.0138 (8)	0.0103 (7)	0.0041 (6)	0.0057 (6)	0.0021 (6)
C8	0.0131 (7)	0.0181 (8)	0.0155 (8)	0.0009 (6)	0.0053 (6)	0.0005 (6)
C9	0.0172 (7)	0.0191 (8)	0.0123 (7)	0.0038 (6)	0.0023 (6)	−0.0025 (6)
C10	0.0262 (8)	0.0205 (9)	0.0119 (7)	0.0066 (7)	0.0097 (7)	0.0016 (6)
C11	0.0255 (9)	0.0229 (9)	0.0202 (9)	−0.0037 (7)	0.0148 (7)	−0.0001 (7)
C12	0.0178 (8)	0.0188 (8)	0.0153 (8)	−0.0036 (6)	0.0070 (6)	−0.0026 (6)
C13	0.0127 (7)	0.0176 (8)	0.0101 (7)	0.0018 (6)	0.0056 (6)	−0.0011 (6)
C14	0.0152 (7)	0.0158 (8)	0.0141 (7)	0.0027 (6)	0.0047 (6)	0.0004 (6)
C15	0.0186 (8)	0.0180 (8)	0.0188 (8)	−0.0004 (6)	0.0078 (7)	−0.0018 (7)
C16	0.0158 (8)	0.0246 (9)	0.0146 (8)	−0.0008 (7)	0.0041 (6)	−0.0043 (7)
C17	0.0161 (8)	0.0245 (9)	0.0128 (8)	0.0057 (7)	0.0037 (6)	0.0006 (6)
C18	0.0161 (7)	0.0177 (8)	0.0139 (7)	0.0044 (6)	0.0066 (6)	0.0011 (6)
C19	0.0109 (7)	0.0133 (8)	0.0098 (7)	−0.0009 (6)	0.0007 (6)	−0.0026 (6)
C20	0.0092 (6)	0.0132 (7)	0.0114 (7)	−0.0010 (6)	0.0027 (6)	−0.0005 (6)
C21	0.0117 (7)	0.0143 (7)	0.0119 (7)	−0.0008 (6)	0.0041 (6)	0.0016 (6)
C22	0.0178 (8)	0.0194 (9)	0.0227 (8)	0.0071 (7)	0.0113 (7)	0.0076 (7)
C23	0.0161 (8)	0.0213 (9)	0.0165 (8)	0.0038 (6)	0.0078 (6)	0.0083 (7)
C24	0.0098 (7)	0.0156 (8)	0.0108 (7)	−0.0023 (6)	0.0035 (6)	−0.0005 (6)
C25	0.0133 (7)	0.0179 (8)	0.0110 (7)	−0.0063 (6)	0.0054 (6)	−0.0031 (6)

### Geometric parameters (Å, °)

**Table:**

Ag1—P1	2.3531 (4)	C8—C9	1.388 (2)
Ag1—O1	2.451 (1)	C8—H8	0.9500
Ag1—O3^i^	2.481 (1)	C9—C10	1.386 (2)
Ag1—N1^ii^	2.253 (1)	C9—H9	0.9500
P1—C1	1.8175 (16)	C10—C11	1.383 (3)
P1—C7	1.8222 (15)	C10—H10	0.9500
P1—C13	1.8248 (17)	C11—C12	1.396 (2)
O1—C19	1.2353 (19)	C11—H11	0.9500
O2—C19	1.2858 (18)	C12—H12	0.9500
O3—C25	1.235 (2)	C13—C18	1.391 (2)
O3—Ag1^iii^	2.4814 (12)	C13—C14	1.404 (2)
O4—C25	1.277 (2)	C14—C15	1.394 (2)
O4—H4	1.20 (4)	C14—H14	0.9500
N1—C21	1.335 (2)	C15—C16	1.390 (2)
N1—C22	1.348 (2)	C15—H15	0.9500
N1—Ag1^ii^	2.2531 (13)	C16—C17	1.386 (2)
C1—C6	1.396 (2)	C16—H16	0.9500
C1—C2	1.397 (2)	C17—C18	1.395 (2)
C2—C3	1.387 (2)	C17—H17	0.9500
C2—H2	0.9500	C18—H18	0.9500
C3—C4	1.387 (2)	C19—C20	1.519 (2)
C3—H3	0.9500	C20—C21	1.397 (2)
C4—C5	1.386 (2)	C20—C24	1.408 (2)
C4—H4A	0.9500	C21—H21	0.9500
C5—C6	1.392 (2)	C22—C23	1.374 (2)
C5—H5	0.9500	C22—H22	0.9500
C6—H6	0.9500	C23—C24	1.395 (2)
C7—C12	1.393 (2)	C23—H23	0.9500
C7—C8	1.398 (2)	C24—C25	1.536 (2)
			
P1—Ag1—O1	113.12 (3)	C9—C10—H10	119.9
P1—Ag1—O3^i^	122.95 (3)	C10—C11—C12	120.18 (16)
P1—Ag1—N1^ii^	140.10 (4)	C10—C11—H11	119.9
O1—Ag1—O3^i^	81.06 (4)	C12—C11—H11	119.9
O1—Ag1—N1^ii^	87.84 (4)	C7—C12—C11	119.94 (16)
O3^i^—Ag1—N1^ii^	92.57 (4)	C7—C12—H12	120.0
C1—P1—C7	104.65 (7)	C11—C12—H12	120.0
C1—P1—C13	104.18 (7)	C18—C13—C14	119.46 (15)
C7—P1—C13	105.28 (7)	C18—C13—P1	122.35 (13)
C1—P1—Ag1	113.79 (5)	C14—C13—P1	118.16 (12)
C7—P1—Ag1	115.70 (5)	C15—C14—C13	119.88 (15)
C13—P1—Ag1	112.16 (5)	C15—C14—H14	120.1
C19—O1—Ag1	123.60 (10)	C13—C14—H14	120.1
C25—O3—Ag1^iii^	132.51 (11)	C16—C15—C14	120.26 (16)
C25—O4—H4	110.1 (17)	C16—C15—H15	119.9
C21—N1—C22	116.87 (14)	C14—C15—H15	119.9
C21—N1—Ag1^ii^	117.57 (10)	C17—C16—C15	119.88 (16)
C22—N1—Ag1^ii^	123.80 (11)	C17—C16—H16	120.1
C6—C1—C2	119.38 (15)	C15—C16—H16	120.1
C6—C1—P1	121.99 (12)	C16—C17—C18	120.31 (16)
C2—C1—P1	118.58 (12)	C16—C17—H17	119.8
C3—C2—C1	120.45 (14)	C18—C17—H17	119.8
C3—C2—H2	119.8	C13—C18—C17	120.19 (16)
C1—C2—H2	119.8	C13—C18—H18	119.9
C2—C3—C4	119.87 (15)	C17—C18—H18	119.9
C2—C3—H3	120.1	O1—C19—O2	122.82 (15)
C4—C3—H3	120.1	O1—C19—C20	117.60 (13)
C5—C4—C3	120.17 (16)	O2—C19—C20	119.58 (14)
C5—C4—H4A	119.9	C21—C20—C24	117.80 (14)
C3—C4—H4A	119.9	C21—C20—C19	113.46 (13)
C4—C5—C6	120.25 (15)	C24—C20—C19	128.70 (14)
C4—C5—H5	119.9	N1—C21—C20	124.81 (14)
C6—C5—H5	119.9	N1—C21—H21	117.6
C5—C6—C1	119.86 (15)	C20—C21—H21	117.6
C5—C6—H6	120.1	N1—C22—C23	122.51 (15)
C1—C6—H6	120.1	N1—C22—H22	118.7
C12—C7—C8	119.29 (14)	C23—C22—H22	118.7
C12—C7—P1	123.54 (12)	C22—C23—C24	121.11 (15)
C8—C7—P1	117.08 (11)	C22—C23—H23	119.4
C9—C8—C7	120.46 (15)	C24—C23—H23	119.4
C9—C8—H8	119.8	C23—C24—C20	116.89 (14)
C7—C8—H8	119.8	C23—C24—C25	115.03 (14)
C10—C9—C8	119.84 (15)	C20—C24—C25	128.06 (14)
C10—C9—H9	120.1	O3—C25—O4	123.62 (15)
C8—C9—H9	120.1	O3—C25—C24	117.47 (14)
C11—C10—C9	120.26 (15)	O4—C25—C24	118.88 (14)
C11—C10—H10	119.9		
			
N1^ii^—Ag1—P1—C1	43.84 (8)	C1—P1—C13—C18	1.57 (15)
O1—Ag1—P1—C1	160.37 (6)	C7—P1—C13—C18	−108.28 (14)
O3^i^—Ag1—P1—C1	−105.31 (7)	Ag1—P1—C13—C18	125.08 (12)
N1^ii^—Ag1—P1—C7	165.10 (8)	C1—P1—C13—C14	−176.52 (12)
O1—Ag1—P1—C7	−78.37 (7)	C7—P1—C13—C14	73.64 (14)
O3^i^—Ag1—P1—C7	15.96 (7)	Ag1—P1—C13—C14	−53.00 (13)
N1^ii^—Ag1—P1—C13	−74.11 (8)	C18—C13—C14—C15	1.6 (2)
O1—Ag1—P1—C13	42.42 (6)	P1—C13—C14—C15	179.71 (12)
O3^i^—Ag1—P1—C13	136.75 (7)	C13—C14—C15—C16	−1.1 (2)
N1^ii^—Ag1—O1—C19	−153.23 (12)	C14—C15—C16—C17	0.1 (3)
P1—Ag1—O1—C19	61.82 (12)	C15—C16—C17—C18	0.4 (3)
O3^i^—Ag1—O1—C19	−60.29 (12)	C14—C13—C18—C17	−1.0 (2)
C7—P1—C1—C6	28.81 (15)	P1—C13—C18—C17	−179.09 (12)
C13—P1—C1—C6	−81.49 (14)	C16—C17—C18—C13	0.1 (2)
Ag1—P1—C1—C6	156.05 (12)	Ag1—O1—C19—O2	−41.8 (2)
C7—P1—C1—C2	−153.99 (12)	Ag1—O1—C19—C20	138.75 (11)
C13—P1—C1—C2	95.70 (13)	O1—C19—C20—C21	12.9 (2)
Ag1—P1—C1—C2	−26.75 (14)	O2—C19—C20—C21	−166.63 (14)
C6—C1—C2—C3	1.3 (2)	O1—C19—C20—C24	−164.64 (15)
P1—C1—C2—C3	−175.96 (12)	O2—C19—C20—C24	15.9 (2)
C1—C2—C3—C4	−0.5 (2)	C22—N1—C21—C20	0.8 (2)
C2—C3—C4—C5	−0.9 (2)	Ag1^ii^—N1—C21—C20	166.32 (12)
C3—C4—C5—C6	1.5 (3)	C24—C20—C21—N1	−0.4 (2)
C4—C5—C6—C1	−0.7 (3)	C19—C20—C21—N1	−178.22 (14)
C2—C1—C6—C5	−0.7 (2)	C21—N1—C22—C23	−0.4 (3)
P1—C1—C6—C5	176.45 (13)	Ag1^ii^—N1—C22—C23	−164.87 (13)
C1—P1—C7—C12	−119.18 (14)	N1—C22—C23—C24	−0.5 (3)
C13—P1—C7—C12	−9.67 (16)	C22—C23—C24—C20	0.9 (2)
Ag1—P1—C7—C12	114.76 (13)	C22—C23—C24—C25	179.83 (15)
C1—P1—C7—C8	64.36 (13)	C21—C20—C24—C23	−0.4 (2)
C13—P1—C7—C8	173.86 (12)	C19—C20—C24—C23	176.96 (15)
Ag1—P1—C7—C8	−61.70 (13)	C21—C20—C24—C25	−179.24 (14)
C12—C7—C8—C9	−0.4 (2)	C19—C20—C24—C25	−1.8 (3)
P1—C7—C8—C9	176.25 (12)	Ag1^iii^—O3—C25—O4	93.68 (18)
C7—C8—C9—C10	−1.0 (2)	Ag1^iii^—O3—C25—C24	−88.43 (17)
C8—C9—C10—C11	1.7 (3)	C23—C24—C25—O3	−13.6 (2)
C9—C10—C11—C12	−1.0 (3)	C20—C24—C25—O3	165.22 (15)
C8—C7—C12—C11	1.1 (2)	C23—C24—C25—O4	164.40 (15)
P1—C7—C12—C11	−175.27 (13)	C20—C24—C25—O4	−16.8 (2)
C10—C11—C12—C7	−0.5 (3)		

Symmetry codes: (i) −*x*+1, *y*−1/2, −*z*+1/2; (ii) −*x*+1, −*y*+1, −*z*+1; (iii) −*x*+1, *y*+1/2, −*z*+1/2.

### Hydrogen-bond geometry (Å, °)

**Table:**

*D*—H···*A*	*D*—H	H···*A*	*D*···*A*	*D*—H···*A*
O4—H4···O2	1.20 (4)	1.20 (4)	2.401 (2)	176 (4)
